# Comparison between Three-Night and Single-Night Actigraphic Sleep Assessment During Early Hospitalization

**DOI:** 10.1055/s-0046-1829644

**Published:** 2026-11-03

**Authors:** Martin Milata, Hana Locihová, Darja Jarošová, Karolína Šrámková, Jana Slonková, Renáta Zoubková, Klára Maternová, Karel Šonka, Marek Susta

**Affiliations:** 1 Department of Neurology and Center of Clinical NeurosciencesFirst Faculty of Medicine, Charles University, General University Hospital in PraguePragueCzech Republic; 2 Department of AnesthesiologyResuscitation and Intensive Care Medicine, Faculty of Medicine, University of Ostrava, Ostrava, Moravian-Silesian RegionCzech Republic; 3 Department of Nursing and MidwiferyFaculty of Medicine, University of Ostrava, Ostrava, Moravian-Silesian RegionCzech Republic; 4 Department of NeurologyUniversity Hospital Ostrava, Ostrava, Moravian-Silesian RegionCzech Republic; 5 Department of Clinical NeurosciencesFaculty of Medicine, University of Ostrava, Ostrava, Moravian-Silesian RegionCzech Republic; 6 Department of AnesthesiologyResuscitation and Intensive Care Medicine, University Hospital Ostrava, Ostrava, Moravian-Silesian RegionCzech Republic; 7 2nd Department of Surgery, Department of Cardiovascular SurgeryFirst Faculty of Medicine, Charles University, General University Hospital in PraguePragueCzech Republic; 8 Science Dynamics Research LabsPragueCzech Republic

**Keywords:** actigraphy, sleep assessment, hospitalization, sleep monitoring, measurement reliability

## Abstract

**Purpose:**

To determine whether a single-night actigraphic sleep assessment during early hospitalization can approximate a 3-night average and characterize inpatient sleep- and rest-activity rhythm.

**Materials and Methods:**

In the present multicenter, cross-sectional study conducted in 7 Czech hospitals, 64 inpatients underwent wrist actigraphic monitoring during the first 3 nights of hospitalization. Actual sleep time, sleep latency, mobile time, sleep efficiency, fragmentation index and average light exposure were analyzed. First-night values were compared with 3-night means using the Wilcoxon test. Individual-level agreement was evaluated using intraclass correlation coefficients (ICCs) and Bland-Altman analysis. Non-parametric circadian rhythm analysis (NPCRA) variables were reported descriptively.

**Results:**

The 3-night mean sleep duration was 342 ± 83 minutes, with reduced sleep efficiency and increased fragmentation. No significant differences were observed between first-night values and 3-night averages for actual sleep time, mobile time, sleep efficiency, fragmentation index or average light exposure. Agreement was good for these parameters (ICC_single ≈ 0.71–0.75), whereas sleep latency showed lower reliability (ICC_single = 0.51). The Bland-Altman analysis demonstrated no systematic bias, with up to 6.3% of observations outside the 95% limits of agreement for most parameters and 9.4% for sleep latency. The NPCRA suggested preserved relative amplitude but fragmented rest-activity rhythm.

**Conclusion:**

Single-night actigraphy provides reliable estimates for most inpatient sleep parameters and may represent a pragmatic alternative to multi-night monitoring in acute hospital settings. Sleep latency is less stable and may require repeated assessment.

## Introduction

Sleep is a fundamental physiological process, and sleep quality significantly influences numerous aspects of life, both in terms of health and disease. Poor sleep lowers the pain threshold, reduces pain tolerance, and increases the need for analgesics. 1 2 It also negatively impacts the incidence of anxiety, depression, and psychotic states, thereby indirectly increasing the use of psychopharmacotherapy, sedation, or other restrictive interventions. Sleep adequacy is reflected in the overall outcomes of hospitalized patients, as its deficiency or reduced quality prolongs recovery time. 3 For these reasons, it is important to assess patient sleep and predict potential disturbances during subsequent nights of hospitalization.

In hospitals, sleep is usually assessed through direct patient questioning or self-reported questionnaires. 4 While questionnaire-based assessment is fast and cost-effective, it is limited by individual interpretation and variability in subjective evaluation. 5 6 The gold standard for objective sleep assessment is polysomnography (PSG); however, despite newer, simplified versions, it remains time-consuming and technically demanding, making it unsuitable for routine use during hospitalization. 7 8

A simpler method, commonly used outside the hospital setting, is actigraphy. This technique records acceleration in three orthogonal planes. Sleep and wake states are inferred from the intensity of movement, providing indirect information on sleep duration, continuity, and nocturnal activity. 9 10 Actigraphy is a non-invasive method that does not disturb the patient and allows for prolonged monitoring in any environment, including during hospitalization.

The present study evaluated whether actigraphic recording during the first night of hospitalization may sufficiently represent a 3-night average for key sleep parameters. A secondary objective was to describe sleep characteristics in acutely hospitalized adults across the first 3 nights of admission.

## Materials and Methods

The current multicenter, cross-sectional observational study was conducted between June 2023 and March 2024 across the internal medicine, surgical, and neurological departments of seven healthcare facilities in the Czech Republic. Actigraphic monitoring was initiated at the start of hospitalization and continued for the first 3 nights. The primary aim was to determine whether actigraphic measurement during the first night of hospitalization sufficiently represents sleep over the first 3 nights. A secondary objective was to describe sleep quality in hospitalized patients. The study was conducted under routine clinical conditions as part of standard care, without any experimental intervention.

A total of 152 patients were enrolled. Inclusion criteria were age over 18 years, hospitalization lasting at least 72 hours in the same department, and signed informed consent. Patients with a Glasgow Coma Scale score below 12 at any point during actigraphic monitoring, terminal-stage illness, a diagnosed sleep disorder, dementia, or significant communication impairment were excluded from the study.

In total, 19 patients were excluded due to technically poor-quality actigraphic recordings, incomplete activity logs, or recordings shorter than 3 consecutive days. An additional 69 patients were excluded due to undergoing surgical procedures, developing delirium, or experiencing changes in or initiation of pharmacotherapy known to affect sleep during monitoring. In total, 88 patients were excluded from the final analysis.

The final analyzed cohort consisted of 64 patients, including 25 men (39.1%), with a mean age of 61.5 ± 16.0 years.

The study used the MotionWatch8 actigraphic device (CamNtech Ltd.) and the proprietary MotionWare software (CamNtech Ltd.), version 1.3.33 for data processing. The actigraph is equipped with a photodiode (lux meter) for quantifying ambient light exposure, which serves to objectively determine the basic parameters of bedtime and wake time.

The recording device was routinely placed on the wrist of the non-dominant upper limb. Movements were recorded in triaxial mode, with activity counts registered in 60-second epochs. The threshold for categorizing an epoch as either rest or movement was set at 20 counts per minute (cpm), the threshold for which the MotionWatch has been validated. 11

Five primary actigraphic parameters were selected for evaluation: actual sleep time, sleep latency, mobile time, sleep efficiency, and fragmentation index, which collectively provide a comprehensive overview of sleep.

1) Actual sleep time (min) is the total time spent sleeping in bed, calculated as the sum of 60-second epochs categorized as rest during the interval between sleep onset and awakening.
2) Sleep latency (min) is the duration between lights out/lying down and the onset of sleep.
3) Mobile time (min) is the total duration of epochs categorized as “with movement” during the sleep period.
4) Sleep efficiency (%) is the ratio of actual sleep time to time spent in bed, expressed as a percentage.
5) Fragmentation index (dimensionless) is defined as the sum of mobile time (%) and immobile bouts ≤ 1 minute (%). Immobile bouts ≤ 1 minute refers to the number of episodes lasting ≤ 1 minute that are categorized as “without movement,” out of the total number of all “without movement” episodes of any duration. 12

Ambient light exposure was also analyzed as an additional actigraphic variable. The average light (lux) parameter represents the average light level during the sleep period, between sleep onset and wake-up time.

In addition to the primary sleep parameters, non-parametric circadian rhythm analysis (NPCRA) was performed to provide a descriptive characterization of the rest-activity rhythm during early hospitalization. The following MotionWare-derived NPCRA variables were extracted:

1) Interdaily stability (IS) quantifies the degree of regularity in the activity-rest pattern with a range of 0 to 1 where a value of 0 indicates a total lack of rhythm and a value of 1 indicates a perfectly stable rhythm.
2) Intradaily variability (IV) quantifies the degree of fragmentation of activity-rest periods. The variable has a theoretical range of 0 to 2 with higher values indicating higher fragmentation. Typical values for healthy subjects will be below 1.
3) Least active 5 hours (L5) average: The L5 average provides the average activity level during the 5 consecutive hours with the lowest activity in the average 24-hour profile, calculated by overlaying and averaging all days of recorded data.
4) L5 onset: Indicates the onset of the L5 window and provides an indication of the phase of the most restful hours.
5) Most active 10 hours(M10) average: The M10 average provides the average activity level during the 10 consecutive hours with the highest activity in the average 24-hour profile, calculated by overlaying and averaging all days of recorded data.
6) M10 onset: Indicates the clock time at which the M10 window begins, indicating the phase of the most active hours.
7) Relative amplitude (RA): (M10 - L5)/(M10 + L5). The variable has a theoretical range of 0 to 1 with higher values indicating a more pronounced day-night activity rhythm. 12

Actigraphic monitoring was conducted continuously over 3 consecutive days and nights. Patient recruitment and actigraph placement within individual departments were performed by nurses specifically trained for the study. Times for going to bed and getting up were recorded manually by patients or staff using a sleep diary.

Entries from the sleep diaries were subsequently verified using recorded ambient light intensity (luxmetry). If the reported lights-out time differed more than once by over 10 minutes from the objectively recorded drop in ambient light levels, the diary entry was considered unreliable, and bedtimes and wake times were determined exclusively based on luxmetry data. The moment of lights-out was defined as the point at which the ambient light intensity declined by at least 50% from the preceding values, and analogously, the time of rising was defined as the moment when the intensity increased by at least 50% compared with previous levels.

Sleep onset time was manually identified from the actigraph's graphical output as the point at which motor activity declined to an individual resting level. This level was defined based on the average activity during a reliably identified, uninterrupted sleep segment lasting at least 10 minutes, verified both anamnestically and through actigraphic parameters.

Wake-up time was defined as the point at which motor activity sustained an increase to a level consistent with wakefulness, lasting at least 10 minutes, and followed by the patient getting out of bed within 60 minutes, without any intervening sleep episode.

Normality was screened visually and with the Kolmogorov-Smirnov test. Because distributions were non-normal, first-night versus 3-night means were compared using the paired Wilcoxon test. Agreement at the individual level was assessed using intraclass correlation coefficients (ICCs) (2-way mixed-effects model, absolute agreement; single- and average-measure ICCs) with 95% confidence intervals. Method-agreement was further examined with Bland-Altman analysis; 95% limits of agreement (LoA) were defined as mean difference ± 1.96 × SD of the differences. The proportion of observations outside the LoA was reported for each parameter. Analyses were performed in IBM SPSS Statistics for Windows (IBM Corp.) software, version 27.0.

Descriptive statistical analysis of the patient cohort was performed, with results presented in the characteristics section. The Kolmogorov-Smirnov test indicated that the data did not follow a normal distribution; therefore, the non-parametric Wilcoxon test was used to assess the statistical significance of differences between the first night and the 3-night average. However, the Wilcoxon test results presented in Table 1 should not be interpreted at the level of individual cases.

**Table 1:** First-night and three-night actigraphy results and their comparisons (paired Wilcoxon test).

Parameter	First night	3-night average	*p* -value
Mean actual sleep time (minutes)	346.0 ± 69.0	342.0 ± 83.0	0.8
Mean sleep latency (minutes)	19.0 ± 25.0	22.0 ± 34.0	0.2
Mean mobile time (minutes)	87.8 ± 50.4	83.3 ± 39.0	0.1
Mean sleep efficiency (%)	72.3 ± 11.5	71.1 ± 14.4	0.9
Mean fragmentation index	41.2 ± 19.0	39.6 ± 22.1	0.2
Average light exposure (lux): median (interquartile range)	1.4 (0.2–6.1)	2.2 (0.7–4.9)	0.8

**Notes:** The average light exposure is presented as median and interquartile range because of its skewed distribution. Differences between first-night values and three-night averages were evaluated using the paired Wilcoxon test.

To evaluate individual-level agreement between single-night and 3-night actigraphic measurements, the ICC was calculated. The value for single measures reflects the reliability of an individual observation, while the value for average measures reflects the reliability of the mean across multiple observations, which reduces variability and improves accuracy. An ICC < 0.5 indicates low reliability; 0.5 to 0.75, moderate reliability; 0.75 to 0.9, good reliability; and > 0.9, excellent reliability. 13

To further assess agreement, Bland-Altman analysis was conducted to complement the ICC. While the ICC quantifies overall consistency, the Bland-Altman method detects systematic bias and individual-level variability. The analysis plots the difference between first-night values and the three-night average against their mean. The key metric is the 95% LoA, which defines the range within which differences between paired measurements are expected to lie with 95% probability. The proportion of outliers—values falling outside the LoA—is the critical indicator of agreement: < 5% is considered high agreement, 5 to 10% acceptable agreement, and > 10% low reliability for substituting one measurement for another. 13

Light exposure was analyzed using the MotionWare *average light (lux)* variable. For each participant, the first-night value was compared with the mean of the first three nights. Because paired differences were not normally distributed, the Wilcoxon signed-rank test was used as the primary paired comparison; paired *t* -test and Spearman correlation were reported as sensitivity and association analyses.

Non-parametric circadian rhythm analysis variables were analyzed descriptively and were not included in the paired 1- versus 3-night comparison. This was because NPCRA parameters are derived from the continuous rest-activity profile over the observation interval, and single-day estimates are methodologically unstable or conceptually inappropriate for selected metrics, particularly interdaily stability.

## Results

During the first 3 nights, patients slept an average of 342 ± 83 minutes, with a mean sleep onset latency of 22 ± 34 minutes. The average mobile time was 83.3 ± 39.0 minutes, and mean sleep efficiency was 71.1% ± 14.4%. The mean fragmentation index was 39.6 ± 22.1. These 3-night averages did not differ significantly from the values obtained on the first night of hospitalization ( Table 1 ).

At the individual level, agreement between 1- and 3-night monitoring was good for most parameters (ICC single > 0.7; *p* < 0.05), indicating solid consistency and reliability. The only exception was sleep latency, in which the agreement was lower (ICC single = 0.51). Differences between single- and multi-night measurements were evenly distributed around zero, indicating no systematic bias. For all parameters except sleep latency, more than 90% of values fell within the 95% LoA, supporting clinical interchangeability between one-night and 3-night recordings. ( Table 2 )

**Table 2:** Individual-level agreement between single-night and 3-night measurements of actigraphic parameters based on the Intraclass Correlation Coefficient (ICC).

Parameter	ICC single measures	ICC average measures	Significance (F test, *p* )
Actual sleep time	0.75; good agreement	0.86; excellent agreement	**< 0.05**
Sleep latency	0.51; fair agreement	0.67; good agreement	**< 0.05**
Mobile time	0.72; good agreement	0.84; excellent agreement	**< 0.05**
Sleep efficiency	0.72; good agreement	0.84; excellent agreement	**< 0.05**
Fragmentation index	0.71; good agreement	0.83; excellent agreement	**< 0.05**

The proportion of outliers—defined as values outside the LoA—was low (3–6%) for actual sleep time, mobile time, sleep efficiency, and fragmentation index, indicating high to good agreement. In contrast, sleep latency showed a higher outlier rate (nearly 10%), consistent with its lower ICC value and confirming that it is a less stable parameter. As such, single-night measurements of sleep latency may not reliably reflect longer-term sleep patterns. ( Table 3 )

**Table 3:** Individual-level agreement between single-night actigraphic measurement and three-night average based on Bland-Altman analysis.

Parameter	Mean difference (single versus mean)	Upper LoA	Lower LoA	Outliers: n (%)
Mean actual sleep time (minutes)	4.3 ± 49.7	101.7	−93.1	**3 (4.7), reliable**
Mean sleep latency (minutes)	−2.1 ± 24.4	45.9	−49.9	**6 (9.4), acceptable**
Mean mobile time (minutes)	3.9 ± 33.8	70.1	−62.3	**4 (6.3), acceptable**
Mean sleep efficiency (%)	0.8 ± 8.5	17.4	−15.8%	**2 (3.1), reliable**
Mean fragmentation index	1.0 ± 13.9	28.2	−26.1	**4 (6.3), acceptable**

**Abbreviations:** LoA, limits of agreement (mean ± 1.96 × SD of differences).

**Note:** Outliers = % of patients outside the LoA.

Average light exposure also did not differ significantly between the first night and the mean of the first 3 nights. ( Table 1 ) The paired difference was not statistically significant (Wilcoxon signed-rank test; *p* = 0.799). First-night and 3-night mean light exposure were strongly correlated (Spearman ρ = 0.714; *p* < 0.001). Bland-Altman analysis showed a mean bias of +0.50 lux, with 95% limits of agreement from −11.35 to +12.34 lux.

Additional NPCRA parameters are summarized in Table 4 . Median L5 average activity was 1,583.6 activity counts (interquartile range [IQR]: 846.9–2,258.0), and median M10 average activity was 13,159.5 activity counts (IQR: 9,056.3–19,954.1). Median RA was 0.8 (IQR: 0.7–0.9), median IS was 0.6 (IQR: 0.5–0.7), and median IV was 1.2 (IQR: 0.9–1.4). The timing of the most active 10-hour period was relatively consistent across participants, with a median M10 start hour of 08:00 (IQR 07:00–10:00). In contrast, the timing of the least active 5-hour period showed greater variability, with a median L5 start hour of 23:00 (IQR 23:00–00:00).

**Table 4:** Non-parametric circadian rhythm analysis parameters.

Parameter	Mean	SD	Median	Q1–Q3*	Minimum–maximum**
NPCRA analysis length, days	3.661	1.346	3.076	3.015–3.751	2.722–10.044
L5 average	1,726.492	1,290.185	1,583.625	846.867–2,258.033	214.000–7,907.867
L5 start hour	11.281	10.973	7.000	0.000–23.000	0.000–23.000
M10 average	14,709.463	8,439.421	13,159.462	9,056.324–19,954.096	1,980.692–43,971.158
M10 start hour	8.531	2.370	8.000	7.000–10.000	3.000–15.000
RA	0.756	0.171	0.808	0.677–0.874	0.119–0.976
IS	0.599	0.145	0.626	0.542–0.698	0.138–0.837
IV	1.165	0.346	1.168	0.899–1.416	0.421–1.865

**Abbreviations** : IS, interdaily stability; IV, intradaily variability; L5, least active 5 hours; M10, most active 10 hours; NPCRA, non-parametric circadian rhythm analysis parameters; RA, relative amplitude.

**Notes** : Interquartile range (Q1–Q3) denotes the range between the first and third quartiles; Minimum–maximum range (min–max) denotes the lowest and highest observed values in the analyzed cohort.

## Discussion

The most important finding of the current study is the high concordance between single-night and 3-nights actigraphic measurements for most sleep actigraphic parameters—actual sleep time, mobile time, sleep efficiency, and fragmentation index—demonstrating the reliability of short-term actigraphic monitoring. The highest reliability was observed for actual sleep time and sleep efficiency. This has practical implications, and the current literature lacks direct comparisons between single- and multi-night actigraphy in both hospital and home settings.

In addition, the observed increase in ICC values for the 3-night averages (except for sleep latency) confirms that greater measurement frequency improves reliability, consistent with previously published findings. 13 The low agreement for sleep latency is not unexpected, as actigraphy-derived sleep onset latency is generally considered a less reliable parameter. 14 Its reduced reliability in the hospital setting is further influenced by situational factors—patients may lie in bed prior to intended sleep onset, and bedtimes may be influenced by staff routines or by other patients in shared rooms.

Our findings also indicate that hospitalized patients experience reduced sleep: the average total sleep time during the first 3 nights was 342 ± 83 minutes (∼ 5.75 hours), which is below the recommended 7 to 9 hours of nightly sleep. 15 Sleep was characterized by frequent nighttime wakefulness, with a mean mobile time of 83.3 ± 39.0 minutes. This corresponds to prolonged wakefulness in bed and reflects increased sleep fragmentation. Physiological values for mobile time are typically < 10% of total sleep time (∼ 34 ± 8 minutes) or < 40 minutes when total sleep exceeds 7 hours—criteria not met in our sample. 9

Sleep efficiency, an overall measure of sleep quality, was reduced (71.1 ± 14.4%) compared with the normal threshold of ≥ 80%. 8 The fragmentation index was elevated 39.6 (±22.1), reflecting nocturnal motor restlessness. This parameter correlates with both sleep efficiency and subjective sleep quality, 16 17 and normal values in healthy individuals are reported as 30 ± 9. 18

The added NPCRA parameters extend these findings from nocturnal sleep to the broader rest-activity profile. The relatively high median RA suggests that the contrast between low-activity and high-activity periods was preserved despite hospitalization. However, this should not be interpreted as evidence of a normal circadian rhythm, because hospital routines and restricted mobility may strongly influence both daytime activity and nocturnal rest. The median IS suggested moderate stability of the rest-activity pattern across the short observation period, potentially reflecting the structured hospital environment, including regular care routines, medication administration, meals, and ward activity. In contrast, the elevated IV indicated fragmentation of the rest-activity rhythm, consistent with the increased nocturnal motor activity and reduced sleep efficiency observed in the primary sleep analysis.

The highly variable L5 start hour is particularly relevant. In non-hospitalized individuals, the least active five-hour period would be expected to occur mainly during the night. In this inpatient cohort, however, L5 timing varied substantially, suggesting that the least active period did not necessarily correspond to biological night sleep. Instead, it may have reflected daytime bed rest, reduced spontaneous mobility, clinical procedures, fatigue, pain, or other hospital-related constraints.

These actigraphy-derived findings confirm the disruption of sleep architecture during hospitalization in standard hospital wards. Reduced sleep duration, elevated mobile time, and a higher fragmentation index all indicate increased motor activity and disturbed sleep. Sleep efficiency below the normative threshold further supports a decline in sleep quality. These observations align with prior studies on hospitalized populations 19 and highlight the importance of good sleep conditions in hospital settings.

Nevertheless, the clinical relevance of reduced sleep duration must be interpreted cautiously. Nocturnal sleep requirements vary individually, and sleep durations below seven hours are not inherently pathological. 20 Although seven hours of nightly sleep is a reasonable clinical target, achieving this in hospitalized patients is often difficult due to environmental and clinical factors, as well as individual sleep variability, especially early in hospitalization.

It is worth considering whether sleep fragmentation, rather than duration alone, has greater clinical impact. Fragmented sleep is associated with lower subjective sleep quality even when total sleep time is preserved. 21 Moreover, disrupted sleep continuity—independent of total duration—has been linked to cognitive decline in middle-aged adults, whereas short sleep duration alone has not. 22

In our study, average sleep latency was 19 minutes, which falls within the normal range in home conditions, generally defined as ≤ 20 minutes, with a slight age-related increase of approximately one minute per decade. 8 This suggests that most patients retained the ability to fall asleep in a standard hospital environment.

Longitudinal actigraphic studies in natural environments indicate that sleep parameters are generally stable over time (i.e., individuals maintain their sleep phenotype). 16 23 24 However, these studies do not address the first-night effect or the influence of unfamiliar environments. In this regard, our study provides novel insights.

The first-night effect has been widely studied in the context of PSG, according to which instrumentation, expectations, and anxiety may interfere with natural sleep patterns. Meta-analyses have demonstrated reduced sleep efficiency, increased wakefulness after sleep onset, delayed rapid eye movement (REM) sleep onset, shortened REM sleep duration, prolonged sleep latency, and decreased total sleep time during the first night of PSG. 25 26

However, sleep disturbances during PSG differ from those encountered during hospitalization. Hospital environments introduce a variety of potential sleep disruptors, including anxiety, pain, concerns about illness, procedural interruptions, environmental noise, light, and room-sharing. While PSG involves physical discomfort and restrictions on movement due to monitoring equipment, it typically lacks the medical stressors and care-related disruptions present in hospital wards. Anxiety is also lower in PSG contexts than during acute hospitalization. 26 27 28

Outside of sleep clinics or diagnostic PSG settings, the first-night effect has been confirmed in hospitalized patients. Studies show decreased total sleep time, increased fragmentation, and lower subjective sleep quality on the first night, with improvements on subsequent nights. The most commonly cited sleep disruptors are noise, light, and nighttime care interventions. 29 30 Improvements over time are likely due to habituation to the hospital environment and stabilization of the clinical condition, both of which may enhance sleep quality.

Because actigraphy is minimally intrusive and often imperceptible to the wearer, it is unlikely to contribute meaningfully to the first-night effect. This finding is supported by a study of 240 healthy volunteers, who were monitored over 5 consecutive nights in their home environment. That study found no significant first-night effect across seven actigraphic parameters, including sleep latency, wake after sleep onset, number of awakenings per hour, time in bed, total sleep time, and sleep efficiency. 31

The strengths of the current study include its multicenter design and the real-world hospital setting, which increases external validity and practical relevance. The large initial sample and consistent 3-night actigraphic monitoring allowed for robust comparisons across time. Patient involvement in sleep diary reporting provided valuable subjective context, and the parallel use of luxmetry enabled verification in cases of uncertainty or limited patient cooperation. The study design adhered to clearly defined eligibility criteria and applied multiple complementary statistical methods, which together strengthened internal consistency and the interpretability of the results.

The selection of patients based on predefined inclusion and exclusion criteria limits the generalizability of the results to the entire population of hospitalized individuals. Moreover, patients who developed significant sleep disturbances during monitoring, which necessitated the initiation of pharmacological sleep interventions, were excluded from the analysis. Consequently, the sample may not fully reflect the most severe degrees of sleep disruption observed in clinical practice. The study population represents clinically stable adult inpatients during early hospitalization. Selection bias toward patients without delirium or major medication changes cannot be excluded. Therefore, generalizability to more severely ill hospitalized populations may be limited. Another limitation concerns the interpretation of NPCRA parameters in the hospital setting. These variables were designed to characterize continuous rest-activity rhythms, but hospitalization may substantially modify spontaneous movement through bed rest, ward routines, clinical procedures, and disease-related immobility. Therefore, NPCRA findings in this study should be interpreted as descriptive inpatient rest-activity characteristics rather than as stable circadian phenotyping. In addition, the short observation period limits the robustness of multi-day rhythm estimates, particularly IS.

Future research should aim to validate the predictive value of actigraphy for key clinical outcomes, such as incidence of delirium, or response to pharmacological and non-pharmacological interventions. Longitudinal studies are needed to assess whether early detection of poor sleep patterns can guide timely interventions and ultimately improve recovery trajectories. Additionally, future work should focus on identifying patient subgroups at highest risk for sleep disruption, optimizing algorithms for automated actigraphy data interpretation, and evaluating the cost-effectiveness and feasibility of implementing single-night actigraphy as part of standard nursing practice. Finally, interventional studies should test targeted strategies for improving sleep in hospitalized patients, including environmental modifications, structured nursing protocols, and personalized behavioral approaches based on actigraphic findings.

## Conclusion

Taken together, the results indicate that 1-night actigraphy provides sufficiently reliable estimates for most sleep parameters and may represent a pragmatic alternative when extended monitoring is not feasible. However, due to its greater intra-individual variability, sleep latency should ideally be assessed over multiple nights.
